# Crystal structure of hexa­kis­(di­methyl­formamide-κ*O*)manganese(II) deca­kis­(di­methyl­formamide)-1κ^5^ *O*,2κ^5^ *O*-[μ-octa­deca­tungstodiphosphato(V)-κ*O*:κ*O*′]dimanganate(II) di­methyl­formamide disolvate

**DOI:** 10.1107/S2056989016007842

**Published:** 2016-05-20

**Authors:** Fatma Dhifallah, Mohamed Salah Belkhiria

**Affiliations:** a Laboratoire de Physico-Chimie des Materiaux, Faculty of Sciences, University of Monastir, Avenue de l’environnement, 5019 Monastir, Tunisia; b University of Sousse, High School of Sciences and Technology, Rue Lamine Abassi, 4011 Hammam Sousse, Tunisia

**Keywords:** crystal structure, polyoxidometalates, organic–inorganic hybrid, Mn^II^ complexes

## Abstract

The crystal structure of the title compound, [Mn(C_3_H_7_NO)_6_][{Mn(C_3_H_7_NO)_5_}_2_(μ-P_2_W_18_O_62_)]·2C_3_H_7_NO, reveals that the Wells–Dawson-type polyanion [P_2_W_18_O_62_]^6−^, through terminal O atoms from the belts, bridges two Mn^II^ octa­hedral complexes. In the complex anion [{Mn(dmf)_5_}_2_(μ-P_2_W_18_O_62_)]^2−^, located around a twofold rotation axis, the Mn^II^ ions are coordinated by five O atoms from di­methyl­formamide (dmf) ligands and to a terminal O atom from the [P_2_W_18_O_62_]^6−^ polyanion. Another Mn^II^ ion, located on an inversion centre, is coordinated by six O atoms from the dmf ligands and forms the [Mn(dmf)_6_]^2+^ cation. The crystal components are connected through numerous weak C—H⋯O hydrogen bonds to construct a three-dimensional network.

## Abstract 2

In the title polyoxidometalate, the Wells–Dawson-type [P_2_W_18_O_62_]^6−^ polyanion bridges two Mn^II^ octa­hedral complexes through terminal O atoms from the belts. The crystal components are connected through numerous weak C—H⋯O hydrogen bonds to construct a three-dimensional framework.

## Chemical context   

Over the past few decades, polyoxidometalates (POMs) have been considered to be inter­esting building blocks for the construction of organic–inorganic hybrid materials because of their nanosize, abundant topologies, controllable shapes and high negative charges (Dolbecq *et al.*, 2010 ▸). As a result of their unique redox, catalytic, photochemical and magnetic properties, organic–inorganic hybrid POM–based materials have captured considerable attention and are applied widely in many fields such as material science, catalysis and medicine (Niu *et al.*, 2004 ▸; Ben Khelifa *et al.*, 2015 ▸). Herein, we report the synthesis, crystal structure and supra­molecular architecture of the title compound, (**1**). 
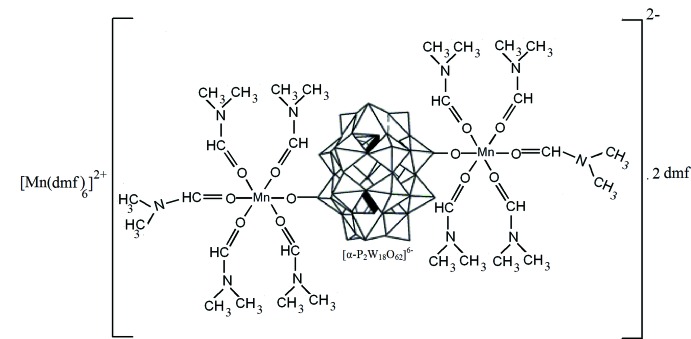


## Structural commentary   

**Figure 1:**
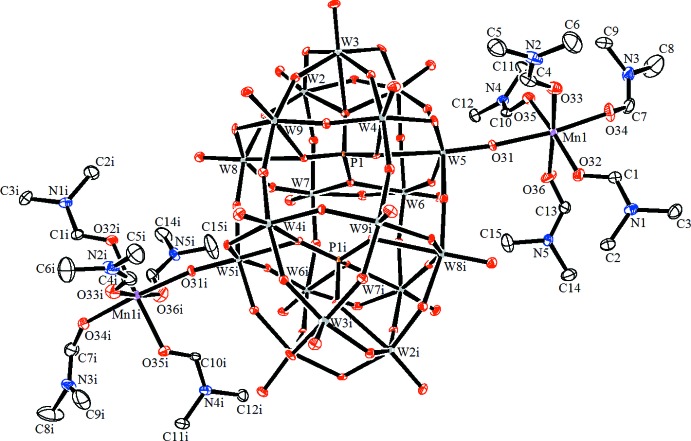
The mol­ecular structure of the anion [{Mn(DMF)_5_}_2_(μ-P_2_W_18_O_62_)]^2−^ in (**1**). Displacement ellipsoids are drawn at the 30% probability level. H atoms have been omitted for clarity. [Symmetry code (i) −*x* + 1, *y*, −*z* + 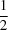.]

**Figure 2:**
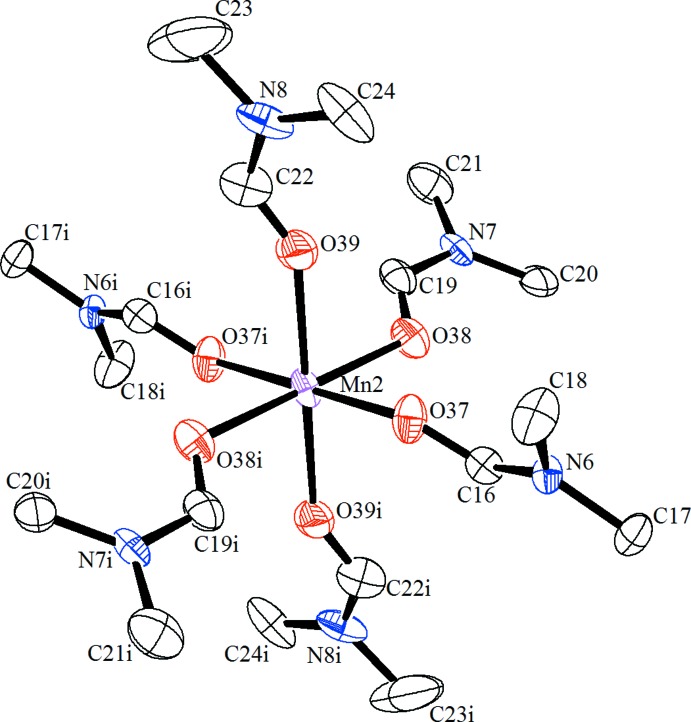
The mol­ecular structure of the [Mn(DMF)_6_]^2+^ cation complex. Displacement ellipsoids are drawn at the 30% probability level. H atoms have been omitted for clarity. [Symmetry code: (i) −*x* + 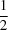
−*y* − 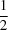, −*z*.]

The asymmetric unit of (**1**) consists of one half of the dinuclear complex anion [{Mn(dmf)_5_}_2_{μ_2_-(α-P_2_W_18_O_62_)}]^2−^, one half of the complex cation [Mn(dmf)_6_]^2+^ and one dmf solvent mol­ecule. The [{Mn(dmf)_5_}_2_{μ_2_-(α-P_2_W_18_O_62_)}]^2−^ anion, with the Wells–Dawson-type polyanion [P_2_W_18_O_62_]^6−^ acting as a bridging ligand between the two Mn^II^ atoms, is located about a twofold symmetry axis (Fig. 1 ▸). The Mn1 ion within this anion is coordinated by five dmf mol­ecules through O atoms and by a terminal O atom from the belt of the Wells–Dawson-type polyanion. The coordination sphere of the Mn1 ion features a trigonal distortion with three shorter [2.137 (6), 2.143 (6) and 2.153 (6) Å] and three longer [2.163 (5), 2.173 (6) and 2.205 (5) Å] Mn1—O bonds. Another Mn^II^ atom in this structure, Mn2, is located on an inversion centre and is octa­hedrally coordinated by six dmf mol­ecules with the formation of the complex cation [Mn(dmf)_6_]^2+^ shown in Fig. 2 ▸. The Mn2 octa­hedron is characterized by a rhombic distortion with the following bond length values: 2.214 (9), 2.175 (7) and 2.134 (7) Å. The O—Mn—O bond angles in the complex ions Mn1 and Mn2 vary from 82.9 (2) to 100.0 (2)° and from 84.2 (3) to 90.6 (3)°, respectively, and from 169.7 (2) to 174.5 (3)° for bond angles with O atoms in *trans* positions in the Mn1 complex. These bond length and angle values show that the octa­hedral coordination geometry of the Mn^II^ ions is distorted and these values are in good agreement with literature data (Niu *et al.*, 2004 ▸).

The two central P atoms of the POM are tetra­hedrally coordinated by four bridging O atoms. The corresponding P1—O bond lengths vary from 1.528 (5) to 1.577 (5) Å (mean value 1.545 Å) and the O—P1—O bond angles are in the range 106.5 (2) to 112.7 (2)°. The W—O distances vary over a wide range: 1.700 (5)–2.384 (4) Å and the bond angles O—W—O are in the range 71.6 (2) to 103.8 (2)°. These bond lengths and angles are consistent with those given in the literature for the α-isomer of the Wells–Dawson-type polyanion (Dhifallah *et al.*, 2016 ▸).

## Supra­molecular features   

**Figure 3:**
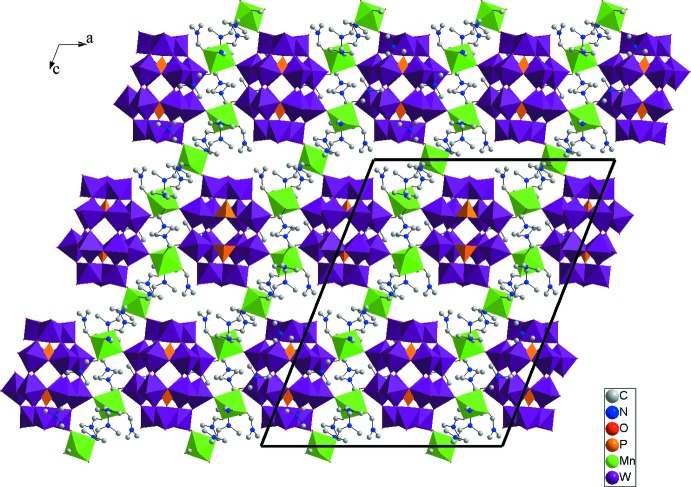
Polyhedral representation of the crystal structure of (**1**), viewed along the *b* axis.

The crystal packing of the title compound is illustrated in Fig. 3 ▸. In addition to the electrostatic inter­actions between the ions, the structure is stabilized by numerous weak C—H⋯O hydrogen bonds (Table 1 ▸) that organize all the structure components into a three-dimensional framework.

## Synthesis and crystallization   

The title compound was prepared at room temperature by dissolving successively the potassium salt K_6_[α-P_2_W_18_O_62_]·11H_2_O (0.606g, 0.125 mmol), synthesized by a literature method (Mbomekalle *et al.*, 2004 ▸) and manganese(II) chloride (MnCl_2_·4H_2_O; 0.099 g, 0.5 mmol) in dmf (25 mL) under stirring. The clear solution obtained was allowed to stand for at least one night until it took a stable color indicating that the kinetics of the reaction were complete. Yellow crystals of (**1**) suitable for X-ray diffraction analysis were obtained by diffusion of ethanol into the dmf solution.

## FT–IR Spectroscopy   

**Figure 4:**
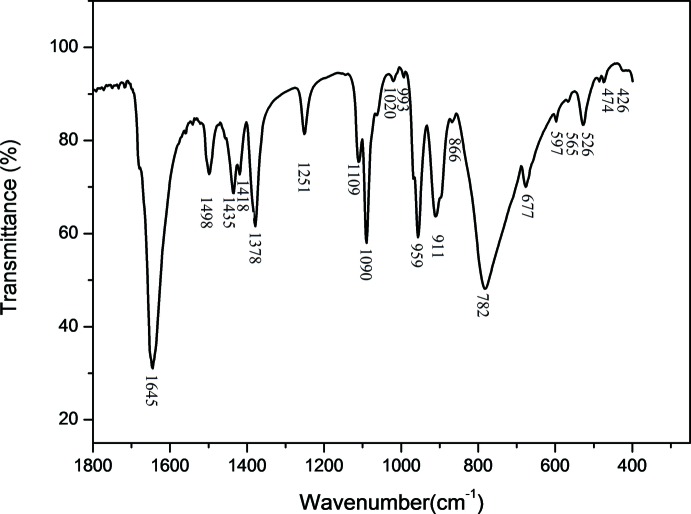
The FT–IR spectrum of (**1**).

The IR spectrum of (**1**) (Fig. 4 ▸) exhibits characteristic bands of metal–oxygen stretching and deformation modes of the POM, in the region 1100–400 cm^−1^. The vibration bands, attributed to ν(P—O_a_), ν(W—O_t_), ν(W—O_e_) and ν(W—O_c_), appear respectively at 1090, 959, 911 and 782 cm^−1^ (Jin *et al.*, 2007 ▸; Dong *et al.*, 2008 ▸; Cao *et al.*, 2009 ▸). The region 1700–1110 cm^−1^ corresponds to DMF mol­ecule vibrations and bands observed at 1645, 1498, 1435, 1418, 1378, 1251 and 1109 cm^−1^ are respectively attributed to ν(CO), δ_as_(CH_3_), δ(CH), δ_s_(CH_3_), ν_as_(C′N) and *r*(CH_3_) (Durgaprasad *et al.*, 1971 ▸).

## Refinement   

Crystal data, data collection and structure refinement details are summarized in Table 2 ▸. All H atoms were refined using a riding model with C—H = 0.93–0.96 Å and *U*_iso_(H) = 1.5*U*_eq_(C) or 1.2*U*_eq_(C). Restraints (DELU and SIMU) in *SHELXL2014* (Sheldrick, 2015 ▸) were used in order to maintain a reasonable geometry and atomic displacement parameters for one DMF mol­ecule.

## Supplementary Material

Crystal structure: contains datablock(s) I. DOI: 10.1107/S2056989016007842/gk2660sup1.cif

Structure factors: contains datablock(s) I. DOI: 10.1107/S2056989016007842/gk2660Isup3.hkl

CCDC reference: 1058770

Additional supporting information:  crystallographic information; 3D view; checkCIF report

## Figures and Tables

**Table 1:** Hydrogen-bond geometry (Å, °)

*D*—H⋯*A*	*D*—H	H⋯*A*	*D*⋯*A*	*D*—H⋯*A*
C1—H1⋯O34	0.93	2.58	3.122 (10)	118
C2—H2*C*⋯O29^i^	0.96	2.61	3.500 (12)	154
C4—H4⋯O21	0.93	2.53	3.431 (11)	163
C4—H4⋯O31	0.93	2.52	3.108 (10)	121
C8—H8*A*⋯O2^ii^	0.96	2.55	3.488 (14)	167
C8—H8*B*⋯O10^iii^	0.96	2.57	3.307 (14)	134
C9—H9*A*⋯O1^ii^	0.96	2.60	3.366 (12)	137
C9—H9*B*⋯O35	0.96	2.42	3.289 (12)	151
C12—H12*A*⋯O23	0.96	2.48	3.439 (9)	176
C12—H12*C*⋯O11	0.96	2.48	3.086 (10)	121
C13—H13⋯O19^iii^	0.93	2.53	3.287 (9)	139
C14—H14*B*⋯O30^i^	0.96	2.56	3.379 (11)	144
C16—H16⋯O10	0.93	2.62	3.473 (11)	153
C17—H17*C*⋯O10	0.96	2.63	3.513 (12)	154
C19—H19⋯O40^iv^	0.93	2.43	3.331 (19)	164
C21—H21*B*⋯O11^ii^	0.96	2.59	3.478 (14)	154
C21—H21*C*⋯O33	0.96	2.50	3.375 (14)	151
C24—H24*C*⋯O26^v^	0.96	2.43	3.369 (14)	164
C25—H25⋯O12^vi^	0.93	2.56	3.358 (19)	144
C26—H26*C*⋯O1^vii^	0.96	2.55	3.453 (17)	156

Symmetry codes: (i) 
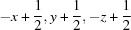
; (ii) 
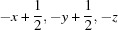
; (iii) 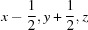; (iv) 
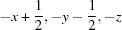
; (v) 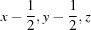; (vi) 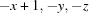; (vii) 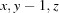.

**Table 2:** Experimental details

Crystal data	
Chemical formula	[Mn(C_3_H_7_NO)_6_][Mn_2_(P_2_W_18_O_62_)(C_3_H_7_NO)_10_]·2C_3_H_7_NO
*M*_r_	5843.78
Crystal system, space group	Monoclinic, *C*2/*c*
Temperature (K)	203
*a*, *b*, *c* (Å)	26.9702 (5), 14.3845 (2), 34.1762 (7)
β (°)	111.896 (2)
*V* (Å^3^)	12302.3 (4)
*Z*	4
Radiation type	Mo *K*α
μ (mm^−1^)	17.18
Crystal size (mm)	0.24 × 0.18 × 0.07

Data collection	
Diffractometer	Agilent SuperNova Dual Source diffractometer with an Atlas detector
Absorption correction	Multi-scan (*CrysAlis PRO*; Agilent, 2014 ▸)
*T*_min_, *T*_max_	0.133, 1.000
No. of measured, independent and observed [*I* > 2σ(*I*)] reflections	110388, 14565, 12616
*R*_int_	0.076
(sin θ/λ)_max_ (Å^−1^)	0.658

Refinement	
*R*[*F* ^2^ > 2σ(*F* ^2^)], *wR*(*F* ^2^), *S*	0.035, 0.072, 1.12
No. of reflections	14565
No. of parameters	804
No. of restraints	7
H-atom treatment	H-atom parameters constrained
Δρ_max_, Δρ_min_ (e Å^−3^)	2.07, −2.08

Computer programs: *CrysAlis PRO* (Agilent, 2014 ▸), *SIR2004* (Burla *et al.*, 2005 ▸), *SHELXL2014* (Sheldrick, 2015 ▸), *ORTEP-3 for Windows* (Farrugia, 2012 ▸) and *DIAMOND* (Brandenburg, 1997 ▸).

## supplementary crystallographic information

### Crystal data

**Table:**

[Mn(C_3_H_7_NO)_6_][Mn_2_(P_2_W_18_O_62_)(C_3_H_7_NO)_10_]·2C_3_H_7_NO	*F*(000) = 10612
*M**_r_* = 5843.78	*D*_x_ = 3.155 Mg m^−^^3^
Monoclinic, *C*2/*c*	Mo *K*α radiation, λ = 0.71073 Å
*a* = 26.9702 (5) Å	Cell parameters from 36494 reflections
*b* = 14.3845 (2) Å	θ = 3.6–29.1°
*c* = 34.1762 (7) Å	µ = 17.18 mm^−^^1^
β = 111.896 (2)°	*T* = 203 K
*V* = 12302.3 (4) Å^3^	Cuboid, yellow
*Z* = 4	0.24 × 0.18 × 0.07 mm

### Data collection

**Table:**

Agilent SuperNova Dual Source diffractometer with an Atlas detector	14565 independent reflections
Radiation source: SuperNova (Mo) X-ray Source	12616 reflections with *I* > 2σ(*I*)
Detector resolution: 5.3048 pixels mm^-1^	*R*_int_ = 0.076
CCD plate, AtlasS2 scans	θ_max_ = 27.9°, θ_min_ = 3.3°
Absorption correction: multi-scan (*CrysAlis PRO*; Agilent, 2014)	*h* = −35→35
*T*_min_ = 0.133, *T*_max_ = 1.000	*k* = −18→18
110388 measured reflections	*l* = −44→44

### Refinement

**Table:**

Refinement on *F*^2^	Primary atom site location: structure-invariant direct methods
Least-squares matrix: full	Secondary atom site location: difference Fourier map
*R*[*F*^2^ > 2σ(*F*^2^)] = 0.035	Hydrogen site location: inferred from neighbouring sites
*wR*(*F*^2^) = 0.072	H-atom parameters constrained
*S* = 1.12	*w* = 1/[σ^2^(*F*_o_^2^) + (0.0162*P*)^2^ + 165.2372*P*] where *P* = (*F*_o_^2^ + 2*F*_c_^2^)/3
14565 reflections	(Δ/σ)_max_ = 0.002
804 parameters	Δρ_max_ = 2.07 e Å^−^^3^
7 restraints	Δρ_min_ = −2.08 e Å^−^^3^

### Special details

**Table:**

Geometry. All esds (except the esd in the dihedral angle between two l.s. planes) are estimated using the full covariance matrix. The cell esds are taken into account individually in the estimation of esds in distances, angles and torsion angles; correlations between esds in cell parameters are only used when they are defined by crystal symmetry. An approximate (isotropic) treatment of cell esds is used for estimating esds involving l.s. planes.

### Fractional atomic coordinates and isotropic or equivalent isotropic displacement parameters (Å^2^)

**Table:**

	*x*	*y*	*z*	*U*_iso_*/*U*_eq_	
W1	0.37578 (2)	0.25005 (2)	0.09160 (2)	0.01620 (6)	
W2	0.50414 (2)	0.24682 (2)	0.09871 (2)	0.01727 (7)	
W3	0.43807 (2)	0.04441 (2)	0.09488 (2)	0.01678 (7)	
W4	0.40732 (2)	−0.02952 (2)	0.18763 (2)	0.01452 (6)	
W5	0.34442 (2)	0.17355 (2)	0.18329 (2)	0.01371 (6)	
W6	0.41338 (2)	0.39654 (2)	0.18805 (2)	0.01413 (6)	
W7	0.54022 (2)	0.39688 (2)	0.19367 (2)	0.01493 (6)	
W8	0.61092 (2)	0.17568 (2)	0.19893 (2)	0.01534 (6)	
W9	0.54669 (2)	−0.02755 (2)	0.19409 (2)	0.01516 (6)	
P1	0.47540 (6)	0.18140 (11)	0.18750 (5)	0.0083 (3)	
Mn2	0.2500	−0.2500	0.0000	0.0409 (5)	
Mn1	0.19102 (4)	0.22616 (8)	0.14462 (3)	0.0211 (2)	
O1	0.43086 (19)	0.2875 (3)	0.07249 (14)	0.0173 (10)	
O2	0.48124 (18)	0.1261 (3)	0.07564 (14)	0.0163 (10)	
O3	0.38000 (18)	0.1277 (3)	0.06966 (14)	0.0185 (11)	
O4	0.39411 (18)	0.3486 (3)	0.13100 (14)	0.0146 (10)	
O5	0.51474 (18)	0.3470 (3)	0.13758 (14)	0.0169 (10)	
O6	0.56452 (19)	0.1928 (3)	0.14063 (15)	0.0182 (10)	
O7	0.50353 (19)	0.0014 (3)	0.13637 (15)	0.0189 (11)	
O8	0.40523 (18)	0.0009 (3)	0.13129 (14)	0.0164 (10)	
O9	0.34566 (18)	0.1923 (3)	0.12803 (14)	0.0163 (10)	
O10	0.4228 (2)	−0.0405 (3)	0.05695 (15)	0.0238 (11)	
O11	0.3207 (2)	0.2898 (3)	0.05154 (15)	0.0235 (11)	
O12	0.5302 (2)	0.2890 (4)	0.06374 (16)	0.0247 (12)	
O13	0.45612 (18)	0.1809 (3)	0.13790 (14)	0.0147 (10)	
O14	0.43198 (18)	0.1303 (3)	0.19850 (13)	0.0130 (9)	
O15	0.52899 (17)	0.1298 (3)	0.20425 (14)	0.0135 (10)	
O16	0.48044 (17)	0.2830 (3)	0.20144 (13)	0.0132 (9)	
O17	0.47296 (19)	0.4582 (3)	0.18215 (14)	0.0189 (11)	
O18	0.59021 (19)	0.2974 (3)	0.20752 (15)	0.0184 (10)	
O19	0.60400 (18)	0.0442 (3)	0.18997 (15)	0.0163 (10)	
O20	0.48063 (18)	−0.0510 (3)	0.20113 (14)	0.0154 (10)	
O21	0.34336 (17)	0.0432 (3)	0.17492 (14)	0.0155 (10)	
O22	0.37225 (18)	0.2954 (3)	0.19530 (14)	0.0149 (10)	
O23	0.36766 (19)	0.4849 (3)	0.17599 (15)	0.0195 (11)	
O24	0.44329 (19)	0.4122 (3)	0.24722 (14)	0.0180 (10)	
O25	0.5769 (2)	0.4870 (3)	0.18580 (15)	0.0227 (11)	
O26	0.6705 (2)	0.1949 (3)	0.19370 (15)	0.0233 (11)	
O27	0.5661 (2)	−0.1362 (3)	0.18549 (16)	0.0248 (12)	
O28	0.58041 (19)	−0.0248 (3)	0.25425 (15)	0.0194 (11)	
O29	0.3805 (2)	−0.1384 (3)	0.17621 (16)	0.0237 (11)	
O30	0.36338 (18)	0.1547 (3)	0.24209 (15)	0.0171 (10)	
O31	0.27687 (18)	0.1915 (3)	0.17009 (15)	0.0196 (11)	
O32	0.1799 (2)	0.1920 (4)	0.20183 (17)	0.0331 (13)	
C1	0.1360 (3)	0.1824 (5)	0.2056 (3)	0.0267 (17)	
H1	0.1054	0.1777	0.1813	0.032*	
N1	0.1307 (3)	0.1788 (4)	0.2425 (2)	0.0292 (15)	
C2	0.1757 (4)	0.1879 (8)	0.2817 (3)	0.051 (3)	
H2A	0.2080	0.1913	0.2761	0.077*	
H2B	0.1773	0.1349	0.2992	0.077*	
H2C	0.1719	0.2434	0.2959	0.077*	
C3	0.0781 (4)	0.1705 (6)	0.2448 (3)	0.039 (2)	
H3A	0.0516	0.1654	0.2168	0.059*	
H3B	0.0710	0.2245	0.2584	0.059*	
H3C	0.0770	0.1161	0.2607	0.059*	
O33	0.1758 (3)	0.0825 (4)	0.12398 (19)	0.0391 (15)	
C4	0.2072 (4)	0.0196 (6)	0.1289 (3)	0.042 (2)	
H4	0.2430	0.0358	0.1368	0.051*	
N2	0.1956 (3)	−0.0703 (5)	0.1241 (2)	0.043 (2)	
C5	0.2378 (4)	−0.1366 (7)	0.1287 (4)	0.061 (3)	
H5A	0.2712	−0.1043	0.1358	0.091*	
H5B	0.2300	−0.1697	0.1027	0.091*	
H5C	0.2403	−0.1798	0.1508	0.091*	
C6	0.1425 (5)	−0.1066 (9)	0.1137 (5)	0.083 (4)	
H6A	0.1202	−0.0598	0.1187	0.124*	
H6B	0.1438	−0.1599	0.1309	0.124*	
H6C	0.1282	−0.1244	0.0845	0.124*	
O34	0.1075 (2)	0.2531 (5)	0.1134 (2)	0.0490 (18)	
C7	0.0757 (4)	0.3129 (8)	0.0919 (3)	0.047 (3)	
H7	0.0626	0.3560	0.1060	0.057*	
N3	0.0595 (3)	0.3190 (6)	0.0512 (2)	0.046 (2)	
C8	0.0175 (6)	0.3811 (11)	0.0260 (4)	0.102 (6)	
H8A	0.0122	0.3740	−0.0032	0.153*	
H8B	−0.0150	0.3665	0.0300	0.153*	
H8C	0.0276	0.4442	0.0345	0.153*	
C9	0.0798 (5)	0.2567 (9)	0.0271 (3)	0.070 (4)	
H9A	0.0634	0.2715	−0.0024	0.105*	
H9B	0.1179	0.2640	0.0361	0.105*	
H9C	0.0717	0.1936	0.0316	0.105*	
O35	0.2046 (2)	0.2857 (4)	0.09136 (17)	0.0309 (13)	
C10	0.2390 (3)	0.3475 (5)	0.0961 (2)	0.0229 (16)	
H10	0.2683	0.3492	0.1214	0.027*	
N4	0.2362 (3)	0.4106 (4)	0.0672 (2)	0.0265 (15)	
C11	0.1928 (4)	0.4060 (7)	0.0266 (3)	0.047 (3)	
H11A	0.1972	0.3522	0.0116	0.071*	
H11B	0.1930	0.4609	0.0107	0.071*	
H11C	0.1595	0.4018	0.0305	0.071*	
C12	0.2799 (3)	0.4762 (5)	0.0728 (3)	0.0312 (19)	
H12A	0.3031	0.4782	0.1020	0.047*	
H12B	0.2655	0.5370	0.0639	0.047*	
H12C	0.2999	0.4564	0.0562	0.047*	
O36	0.2124 (3)	0.3636 (4)	0.1703 (2)	0.0421 (16)	
C13	0.2043 (3)	0.4050 (6)	0.1989 (3)	0.036 (2)	
H13	0.1689	0.4132	0.1960	0.043*	
N5	0.2409 (3)	0.4385 (5)	0.2327 (2)	0.0294 (15)	
C14	0.2278 (4)	0.4882 (8)	0.2646 (3)	0.056 (3)	
H14A	0.2602	0.5071	0.2870	0.084*	
H14B	0.2069	0.5421	0.2522	0.084*	
H14C	0.2078	0.4483	0.2758	0.084*	
C15	0.2964 (4)	0.4234 (11)	0.2408 (4)	0.092 (5)	
H15A	0.3123	0.3885	0.2663	0.138*	
H15B	0.3142	0.4823	0.2436	0.138*	
H15C	0.2999	0.3895	0.2177	0.138*	
O37	0.3191 (3)	−0.2804 (5)	0.0546 (2)	0.058 (2)	
C16	0.3642 (4)	−0.2458 (7)	0.0716 (3)	0.045 (2)	
H16	0.3728	−0.1956	0.0581	0.054*	
N6	0.4007 (3)	−0.2743 (5)	0.1069 (2)	0.0357 (17)	
C17	0.4532 (4)	−0.2344 (7)	0.1243 (3)	0.053 (3)	
H17A	0.4794	−0.2818	0.1272	0.080*	
H17B	0.4589	−0.2082	0.1515	0.080*	
H17C	0.4565	−0.1865	0.1058	0.080*	
C18	0.3876 (5)	−0.3450 (8)	0.1311 (4)	0.070 (4)	
H18A	0.3500	−0.3577	0.1190	0.106*	
H18B	0.3970	−0.3240	0.1596	0.106*	
H18C	0.4071	−0.4007	0.1309	0.106*	
O38	0.2501 (3)	−0.1093 (5)	0.0233 (2)	0.058 (2)	
C19	0.2095 (5)	−0.0671 (8)	0.0183 (3)	0.054 (3)	
H19	0.1771	−0.0971	0.0045	0.065*	
N7	0.2083 (4)	0.0211 (5)	0.0315 (2)	0.044 (2)	
C20	0.2519 (6)	0.0778 (11)	0.0525 (5)	0.104 (6)	
H20A	0.2840	0.0413	0.0617	0.156*	
H20B	0.2474	0.1056	0.0765	0.156*	
H20C	0.2543	0.1257	0.0337	0.156*	
C21	0.1563 (6)	0.0636 (9)	0.0208 (4)	0.079 (4)	
H21A	0.1290	0.0182	0.0079	0.119*	
H21B	0.1526	0.1140	0.0016	0.119*	
H21C	0.1527	0.0868	0.0460	0.119*	
O39	0.2057 (3)	−0.2996 (5)	0.0390 (3)	0.067 (2)	
C22	0.1679 (6)	−0.3436 (8)	0.0404 (4)	0.069 (3)	
H22	0.1387	−0.3512	0.0153	0.083*	
N8	0.1648 (5)	−0.3803 (7)	0.0733 (3)	0.067 (3)	
C23	0.1136 (8)	−0.4306 (18)	0.0683 (8)	0.184 (11)	
H23A	0.1160	−0.4551	0.0951	0.276*	
H23B	0.0842	−0.3879	0.0581	0.276*	
H23C	0.1081	−0.4806	0.0485	0.276*	
C24	0.2036 (7)	−0.3869 (10)	0.1143 (4)	0.096 (5)	
H24A	0.2367	−0.3613	0.1148	0.144*	
H24B	0.2087	−0.4510	0.1227	0.144*	
H24C	0.1918	−0.3529	0.1334	0.144*	
O40	0.4111 (6)	−0.3352 (9)	0.0133 (4)	0.136 (5)	
C25	0.4542 (6)	−0.3711 (11)	0.0229 (6)	0.109 (6)	
H25	0.4747	−0.3531	0.0076	0.131*	
N9	0.4758 (5)	−0.4331 (10)	0.0530 (5)	0.111 (5)	
C26	0.4464 (9)	−0.4739 (11)	0.0759 (5)	0.125 (7)	
H26A	0.4150	−0.4376	0.0717	0.188*	
H26B	0.4684	−0.4754	0.1054	0.188*	
H26C	0.4362	−0.5361	0.0660	0.188*	
C27	0.5270 (9)	−0.4643 (16)	0.0653 (11)	0.27 (2)	
H27A	0.5304	−0.5217	0.0804	0.410*	
H27B	0.5508	−0.4189	0.0833	0.410*	
H27C	0.5359	−0.4743	0.0409	0.410*	

### Atomic displacement parameters (Å^2^)

**Table:**

	*U*^11^	*U*^22^	*U*^33^	*U*^12^	*U*^13^	*U*^23^
W1	0.01490 (14)	0.02190 (14)	0.01007 (13)	0.00222 (11)	0.00265 (11)	0.00061 (10)
W2	0.01742 (15)	0.02454 (15)	0.01067 (13)	−0.00453 (12)	0.00617 (11)	−0.00034 (11)
W3	0.02004 (15)	0.01751 (13)	0.01187 (14)	−0.00120 (11)	0.00488 (11)	−0.00361 (10)
W4	0.01286 (14)	0.01258 (13)	0.01811 (14)	−0.00222 (10)	0.00579 (11)	−0.00138 (10)
W5	0.00908 (13)	0.01626 (13)	0.01540 (14)	0.00011 (10)	0.00411 (11)	0.00085 (10)
W6	0.01424 (14)	0.01239 (13)	0.01491 (14)	0.00162 (10)	0.00446 (11)	0.00112 (10)
W7	0.01552 (14)	0.01431 (13)	0.01575 (14)	−0.00319 (11)	0.00675 (11)	0.00008 (10)
W8	0.01178 (14)	0.02008 (14)	0.01606 (14)	−0.00088 (11)	0.00738 (11)	−0.00110 (10)
W9	0.01322 (14)	0.01455 (13)	0.01807 (15)	0.00186 (10)	0.00625 (11)	−0.00268 (10)
P1	0.0058 (8)	0.0100 (7)	0.0089 (8)	0.0000 (6)	0.0025 (6)	0.0000 (6)
Mn2	0.0532 (13)	0.0319 (10)	0.0293 (10)	0.0081 (9)	0.0059 (9)	0.0001 (8)
Mn1	0.0162 (6)	0.0282 (6)	0.0178 (6)	0.0026 (5)	0.0052 (4)	0.0002 (4)
O1	0.018 (3)	0.024 (2)	0.008 (2)	−0.002 (2)	0.0035 (19)	0.0029 (19)
O2	0.018 (3)	0.018 (2)	0.016 (2)	0.002 (2)	0.009 (2)	−0.0013 (19)
O3	0.012 (2)	0.025 (3)	0.012 (2)	−0.002 (2)	−0.0043 (19)	−0.0049 (19)
O4	0.015 (2)	0.016 (2)	0.012 (2)	0.0017 (19)	0.0049 (19)	0.0009 (18)
O5	0.016 (2)	0.022 (2)	0.013 (2)	−0.002 (2)	0.006 (2)	0.0003 (19)
O6	0.018 (3)	0.022 (2)	0.015 (2)	−0.001 (2)	0.007 (2)	−0.0022 (19)
O7	0.019 (3)	0.020 (2)	0.017 (3)	0.003 (2)	0.007 (2)	−0.0027 (19)
O8	0.014 (2)	0.020 (2)	0.014 (2)	−0.0013 (19)	0.0036 (19)	−0.0015 (19)
O9	0.015 (2)	0.020 (2)	0.013 (2)	−0.0005 (19)	0.0044 (19)	0.0004 (19)
O10	0.029 (3)	0.023 (3)	0.019 (3)	−0.003 (2)	0.010 (2)	−0.009 (2)
O11	0.019 (3)	0.029 (3)	0.018 (3)	0.003 (2)	0.002 (2)	0.001 (2)
O12	0.019 (3)	0.036 (3)	0.022 (3)	−0.006 (2)	0.011 (2)	0.002 (2)
O13	0.015 (2)	0.018 (2)	0.012 (2)	−0.0001 (19)	0.0053 (19)	−0.0004 (18)
O14	0.017 (2)	0.012 (2)	0.010 (2)	0.0009 (18)	0.0064 (19)	−0.0024 (17)
O15	0.013 (2)	0.013 (2)	0.013 (2)	−0.0034 (18)	0.0029 (19)	−0.0026 (18)
O16	0.014 (2)	0.018 (2)	0.008 (2)	0.0017 (19)	0.0044 (18)	0.0029 (18)
O17	0.024 (3)	0.016 (2)	0.015 (2)	−0.001 (2)	0.005 (2)	0.0035 (19)
O18	0.020 (3)	0.021 (2)	0.016 (2)	−0.002 (2)	0.009 (2)	0.0021 (19)
O19	0.009 (2)	0.020 (2)	0.022 (3)	0.0038 (19)	0.008 (2)	0.0028 (19)
O20	0.013 (2)	0.016 (2)	0.018 (2)	−0.0002 (19)	0.0061 (19)	0.0010 (19)
O21	0.009 (2)	0.015 (2)	0.021 (3)	−0.0022 (18)	0.0035 (19)	0.0048 (19)
O22	0.013 (2)	0.017 (2)	0.012 (2)	0.0032 (19)	0.0019 (19)	0.0007 (18)
O23	0.021 (3)	0.019 (2)	0.016 (3)	0.004 (2)	0.005 (2)	0.0037 (19)
O24	0.019 (3)	0.016 (2)	0.018 (3)	−0.001 (2)	0.007 (2)	−0.0009 (19)
O25	0.026 (3)	0.022 (3)	0.023 (3)	−0.008 (2)	0.012 (2)	0.001 (2)
O26	0.021 (3)	0.032 (3)	0.019 (3)	−0.002 (2)	0.009 (2)	−0.002 (2)
O27	0.028 (3)	0.022 (3)	0.022 (3)	0.007 (2)	0.006 (2)	−0.006 (2)
O28	0.017 (3)	0.022 (3)	0.019 (3)	−0.001 (2)	0.006 (2)	−0.002 (2)
O29	0.029 (3)	0.017 (2)	0.025 (3)	−0.007 (2)	0.011 (2)	−0.005 (2)
O30	0.013 (2)	0.022 (2)	0.019 (3)	−0.0005 (19)	0.009 (2)	0.0016 (19)
O31	0.011 (2)	0.025 (3)	0.022 (3)	−0.001 (2)	0.004 (2)	−0.001 (2)
O32	0.028 (3)	0.052 (4)	0.021 (3)	0.006 (3)	0.012 (2)	0.007 (3)
C1	0.032 (5)	0.023 (4)	0.025 (4)	−0.001 (3)	0.011 (4)	0.001 (3)
N1	0.037 (4)	0.029 (4)	0.022 (4)	0.002 (3)	0.012 (3)	0.002 (3)
C2	0.043 (6)	0.090 (8)	0.023 (5)	−0.001 (5)	0.014 (4)	−0.008 (5)
C3	0.044 (6)	0.043 (5)	0.042 (6)	−0.004 (4)	0.028 (5)	0.001 (4)
O33	0.045 (4)	0.032 (3)	0.036 (4)	−0.003 (3)	0.010 (3)	−0.004 (3)
C4	0.043 (6)	0.042 (5)	0.033 (5)	−0.008 (5)	0.004 (4)	0.000 (4)
N2	0.055 (5)	0.029 (4)	0.031 (4)	−0.010 (4)	−0.001 (4)	0.000 (3)
C5	0.057 (7)	0.048 (6)	0.068 (8)	−0.006 (5)	0.013 (6)	−0.008 (6)
C6	0.062 (8)	0.079 (9)	0.103 (11)	−0.028 (7)	0.025 (8)	−0.020 (8)
O34	0.021 (3)	0.084 (5)	0.040 (4)	0.005 (3)	0.010 (3)	0.027 (4)
C7	0.037 (6)	0.074 (7)	0.040 (6)	0.013 (5)	0.023 (5)	0.005 (5)
N3	0.028 (4)	0.075 (6)	0.032 (4)	0.019 (4)	0.008 (3)	0.008 (4)
C8	0.100 (11)	0.145 (14)	0.060 (9)	0.082 (11)	0.029 (8)	0.041 (9)
C9	0.053 (7)	0.101 (10)	0.047 (7)	−0.004 (7)	0.008 (6)	−0.040 (7)
O35	0.031 (3)	0.038 (3)	0.023 (3)	−0.006 (3)	0.010 (2)	0.004 (2)
C10	0.024 (4)	0.031 (4)	0.011 (4)	0.002 (3)	0.004 (3)	0.002 (3)
N4	0.030 (4)	0.029 (3)	0.020 (3)	0.006 (3)	0.008 (3)	0.003 (3)
C11	0.043 (6)	0.065 (7)	0.025 (5)	−0.010 (5)	0.002 (4)	0.013 (4)
C12	0.026 (4)	0.028 (4)	0.036 (5)	0.000 (3)	0.008 (4)	0.005 (4)
O36	0.056 (4)	0.037 (3)	0.044 (4)	−0.001 (3)	0.030 (3)	−0.015 (3)
C13	0.027 (5)	0.039 (5)	0.038 (5)	0.007 (4)	0.008 (4)	−0.011 (4)
N5	0.022 (4)	0.033 (4)	0.033 (4)	0.009 (3)	0.010 (3)	−0.004 (3)
C14	0.051 (7)	0.079 (8)	0.036 (6)	0.020 (6)	0.012 (5)	−0.015 (5)
C15	0.028 (6)	0.140 (13)	0.097 (11)	0.004 (7)	0.012 (6)	−0.046 (10)
O37	0.056 (5)	0.059 (5)	0.040 (4)	−0.004 (4)	−0.002 (4)	0.003 (3)
C16	0.053 (7)	0.039 (5)	0.044 (6)	0.003 (5)	0.020 (5)	0.003 (4)
N6	0.043 (5)	0.029 (4)	0.030 (4)	−0.008 (3)	0.009 (3)	0.004 (3)
C17	0.038 (6)	0.049 (6)	0.065 (7)	−0.008 (5)	0.012 (5)	−0.003 (5)
C18	0.073 (8)	0.065 (7)	0.060 (8)	−0.021 (6)	0.010 (6)	0.031 (6)
O38	0.076 (6)	0.047 (4)	0.051 (5)	0.008 (4)	0.024 (4)	−0.003 (3)
C19	0.077 (8)	0.053 (7)	0.038 (6)	−0.006 (6)	0.029 (6)	0.002 (5)
N7	0.072 (6)	0.032 (4)	0.036 (5)	0.000 (4)	0.030 (4)	−0.004 (3)
C20	0.112 (12)	0.141 (13)	0.099 (11)	−0.079 (11)	0.086 (10)	−0.065 (10)
C21	0.099 (11)	0.088 (10)	0.052 (8)	0.020 (8)	0.031 (7)	0.012 (7)
O39	0.077 (6)	0.051 (5)	0.086 (6)	−0.010 (4)	0.045 (5)	−0.021 (4)
C22	0.081 (9)	0.061 (7)	0.071 (8)	0.021 (6)	0.035 (7)	0.017 (6)
N8	0.092 (8)	0.076 (7)	0.053 (6)	0.032 (6)	0.049 (6)	0.018 (5)
C23	0.098 (15)	0.28 (3)	0.22 (3)	0.007 (18)	0.110 (17)	−0.01 (2)
C24	0.162 (16)	0.078 (9)	0.054 (9)	0.028 (10)	0.048 (10)	−0.009 (7)
O40	0.129 (11)	0.134 (10)	0.145 (11)	0.037 (9)	0.051 (9)	0.054 (9)
C25	0.046 (8)	0.098 (11)	0.190 (19)	0.031 (8)	0.051 (10)	0.063 (12)
N9	0.083 (10)	0.097 (10)	0.136 (13)	0.024 (8)	0.021 (9)	0.022 (9)
C26	0.22 (2)	0.088 (11)	0.069 (10)	0.044 (13)	0.060 (12)	0.050 (9)
C27	0.096 (17)	0.14 (2)	0.55 (6)	0.046 (15)	0.08 (3)	0.14 (3)

### Geometric parameters (Å, º)

**Table:**

W1—O11	1.700 (5)	C4—H4	0.9300
W1—O4	1.890 (4)	N2—C6	1.439 (13)
W1—O1	1.910 (5)	N2—C5	1.448 (13)
W1—O9	1.912 (5)	C5—H5A	0.9600
W1—O3	1.933 (5)	C5—H5B	0.9600
W1—O13	2.373 (4)	C5—H5C	0.9600
W2—O12	1.708 (5)	C6—H6A	0.9600
W2—O6	1.889 (5)	C6—H6B	0.9600
W2—O5	1.908 (5)	C6—H6C	0.9600
W2—O2	1.912 (4)	O34—C7	1.243 (11)
W2—O1	1.932 (5)	C7—N3	1.296 (12)
W2—O13	2.380 (5)	C7—H7	0.9300
W3—O10	1.715 (5)	N3—C8	1.447 (13)
W3—O8	1.883 (5)	N3—C9	1.458 (13)
W3—O3	1.904 (5)	C8—H8A	0.9600
W3—O7	1.909 (5)	C8—H8B	0.9600
W3—O2	1.934 (5)	C8—H8C	0.9600
W3—O13	2.392 (4)	C9—H9A	0.9600
W4—O29	1.707 (5)	C9—H9B	0.9600
W4—O20	1.881 (4)	C9—H9C	0.9600
W4—O28^i^	1.889 (5)	O35—C10	1.252 (9)
W4—O21	1.924 (4)	C10—N4	1.321 (9)
W4—O8	1.955 (5)	C10—H10	0.9300
W4—O14	2.384 (4)	N4—C11	1.445 (10)
W5—O31	1.725 (5)	N4—C12	1.467 (10)
W5—O22	1.890 (4)	C11—H11A	0.9600
W5—O21	1.895 (4)	C11—H11B	0.9600
W5—O30	1.899 (5)	C11—H11C	0.9600
W5—O9	1.920 (5)	C12—H12A	0.9600
W5—O14	2.306 (4)	C12—H12B	0.9600
W6—O23	1.711 (5)	C12—H12C	0.9600
W6—O24	1.891 (5)	O36—C13	1.230 (10)
W6—O22	1.901 (5)	C13—N5	1.301 (10)
W6—O17	1.911 (5)	C13—H13	0.9300
W6—O4	1.947 (4)	N5—C15	1.434 (12)
W6—O16	2.352 (4)	N5—C14	1.453 (11)
W7—O25	1.711 (5)	C14—H14A	0.9600
W7—O18	1.901 (5)	C14—H14B	0.9600
W7—O24^i^	1.912 (5)	C14—H14C	0.9600
W7—O5	1.918 (5)	C15—H15A	0.9600
W7—O17	1.921 (5)	C15—H15B	0.9600
W7—O16	2.381 (4)	C15—H15C	0.9600
W8—O26	1.704 (5)	O37—C16	1.241 (11)
W8—O18	1.894 (5)	C16—N6	1.306 (11)
W8—O30^i^	1.895 (5)	C16—H16	0.9300
W8—O19	1.913 (5)	N6—C18	1.435 (11)
W8—O6	1.933 (5)	N6—C17	1.436 (11)
W8—O15	2.377 (4)	C17—H17A	0.9600
W9—O27	1.709 (5)	C17—H17B	0.9600
W9—O19	1.907 (5)	C17—H17C	0.9600
W9—O28	1.913 (5)	C18—H18A	0.9600
W9—O20	1.915 (4)	C18—H18B	0.9600
W9—O7	1.925 (5)	C18—H18C	0.9600
W9—O15	2.365 (4)	O38—C19	1.208 (13)
P1—O16	1.528 (5)	C19—N7	1.350 (12)
P1—O15	1.533 (5)	C19—H19	0.9300
P1—O14	1.543 (5)	N7—C20	1.389 (14)
P1—O13	1.577 (5)	N7—C21	1.448 (15)
Mn2—O37^ii^	2.133 (7)	C20—H20A	0.9600
Mn2—O37	2.134 (7)	C20—H20B	0.9600
Mn2—O38^ii^	2.175 (7)	C20—H20C	0.9600
Mn2—O38	2.175 (7)	C21—H21A	0.9600
Mn2—O39^ii^	2.214 (9)	C21—H21B	0.9600
Mn2—O39	2.214 (9)	C21—H21C	0.9600
Mn1—O34	2.137 (6)	O39—C22	1.216 (14)
Mn1—O32	2.143 (6)	C22—N8	1.272 (14)
Mn1—O36	2.153 (6)	C22—H22	0.9300
Mn1—O35	2.163 (5)	N8—C24	1.404 (16)
Mn1—O33	2.173 (6)	N8—C23	1.51 (2)
Mn1—O31	2.205 (5)	C23—H23A	0.9600
O24—W7^i^	1.912 (5)	C23—H23B	0.9600
O28—W4^i^	1.889 (5)	C23—H23C	0.9600
O30—W8^i^	1.895 (5)	C24—H24A	0.9600
O32—C1	1.245 (9)	C24—H24B	0.9600
C1—N1	1.322 (10)	C24—H24C	0.9600
C1—H1	0.9300	O40—C25	1.201 (16)
N1—C2	1.439 (11)	C25—N9	1.320 (19)
N1—C3	1.454 (11)	C25—H25	0.9300
C2—H2A	0.9600	N9—C27	1.36 (2)
C2—H2B	0.9600	N9—C26	1.43 (2)
C2—H2C	0.9600	C26—H26A	0.9600
C3—H3A	0.9600	C26—H26B	0.9600
C3—H3B	0.9600	C26—H26C	0.9600
C3—H3C	0.9600	C27—H27A	0.9600
O33—C4	1.207 (11)	C27—H27B	0.9600
C4—N2	1.325 (11)	C27—H27C	0.9600
			
O11—W1—O4	103.8 (2)	W5—O14—W4	91.28 (16)
O11—W1—O1	100.8 (2)	P1—O15—W9	128.5 (2)
O4—W1—O1	90.1 (2)	P1—O15—W8	127.6 (2)
O11—W1—O9	102.4 (2)	W9—O15—W8	90.60 (16)
O4—W1—O9	85.64 (19)	P1—O16—W6	129.1 (3)
O1—W1—O9	156.78 (19)	P1—O16—W7	127.5 (3)
O11—W1—O3	98.9 (2)	W6—O16—W7	90.25 (15)
O4—W1—O3	157.21 (18)	W6—O17—W7	122.1 (2)
O1—W1—O3	87.6 (2)	W8—O18—W7	151.7 (3)
O9—W1—O3	87.6 (2)	W9—O19—W8	123.8 (2)
O11—W1—O13	169.8 (2)	W4—O20—W9	151.9 (3)
O4—W1—O13	84.73 (17)	W5—O21—W4	122.8 (2)
O1—W1—O13	73.32 (17)	W5—O22—W6	152.2 (3)
O9—W1—O13	83.55 (18)	W6—O24—W7^i^	162.7 (3)
O3—W1—O13	72.89 (16)	W4^i^—O28—W9	162.9 (3)
O12—W2—O6	102.3 (2)	W8^i^—O30—W5	161.8 (3)
O12—W2—O5	103.0 (2)	W5—O31—Mn1	171.1 (3)
O6—W2—O5	85.4 (2)	C1—O32—Mn1	125.6 (5)
O12—W2—O2	100.9 (2)	O32—C1—N1	123.3 (8)
O6—W2—O2	89.63 (19)	O32—C1—H1	118.4
O5—W2—O2	156.2 (2)	N1—C1—H1	118.4
O12—W2—O1	100.6 (2)	C1—N1—C2	122.0 (8)
O6—W2—O1	157.0 (2)	C1—N1—C3	120.6 (7)
O5—W2—O1	87.5 (2)	C2—N1—C3	117.3 (7)
O2—W2—O1	88.1 (2)	N1—C2—H2A	109.5
O12—W2—O13	171.0 (2)	N1—C2—H2B	109.5
O6—W2—O13	84.57 (18)	H2A—C2—H2B	109.5
O5—W2—O13	83.16 (18)	N1—C2—H2C	109.5
O2—W2—O13	73.16 (17)	H2A—C2—H2C	109.5
O1—W2—O13	72.82 (17)	H2B—C2—H2C	109.5
O10—W3—O8	102.9 (2)	N1—C3—H3A	109.5
O10—W3—O3	100.4 (2)	N1—C3—H3B	109.5
O8—W3—O3	89.9 (2)	H3A—C3—H3B	109.5
O10—W3—O7	103.0 (2)	N1—C3—H3C	109.5
O8—W3—O7	86.2 (2)	H3A—C3—H3C	109.5
O3—W3—O7	156.52 (19)	H3B—C3—H3C	109.5
O10—W3—O2	100.4 (2)	C4—O33—Mn1	129.1 (6)
O8—W3—O2	156.61 (19)	O33—C4—N2	126.4 (9)
O3—W3—O2	87.8 (2)	O33—C4—H4	116.8
O7—W3—O2	86.7 (2)	N2—C4—H4	116.8
O10—W3—O13	170.2 (2)	C4—N2—C6	123.5 (10)
O8—W3—O13	84.54 (18)	C4—N2—C5	119.3 (9)
O3—W3—O13	72.92 (17)	C6—N2—C5	117.2 (9)
O7—W3—O13	83.66 (17)	N2—C5—H5A	109.5
O2—W3—O13	72.54 (17)	N2—C5—H5B	109.5
O29—W4—O20	102.8 (2)	H5A—C5—H5B	109.5
O29—W4—O28^i^	99.6 (2)	N2—C5—H5C	109.5
O20—W4—O28^i^	89.5 (2)	H5A—C5—H5C	109.5
O29—W4—O21	100.4 (2)	H5B—C5—H5C	109.5
O20—W4—O21	156.48 (19)	N2—C6—H6A	109.5
O28^i^—W4—O21	90.5 (2)	N2—C6—H6B	109.5
O29—W4—O8	97.2 (2)	H6A—C6—H6B	109.5
O20—W4—O8	85.41 (19)	N2—C6—H6C	109.5
O28^i^—W4—O8	163.13 (19)	H6A—C6—H6C	109.5
O21—W4—O8	87.81 (19)	H6B—C6—H6C	109.5
O29—W4—O14	171.8 (2)	C7—O34—Mn1	142.0 (7)
O20—W4—O14	85.05 (17)	O34—C7—N3	124.3 (10)
O28^i^—W4—O14	82.65 (18)	O34—C7—H7	117.9
O21—W4—O14	71.64 (17)	N3—C7—H7	117.9
O8—W4—O14	80.89 (17)	C7—N3—C8	123.8 (9)
O31—W5—O22	102.3 (2)	C7—N3—C9	121.3 (9)
O31—W5—O21	98.8 (2)	C8—N3—C9	114.7 (9)
O22—W5—O21	158.81 (19)	N3—C8—H8A	109.5
O31—W5—O30	98.0 (2)	N3—C8—H8B	109.5
O22—W5—O30	88.52 (19)	H8A—C8—H8B	109.5
O21—W5—O30	90.1 (2)	N3—C8—H8C	109.5
O31—W5—O9	97.2 (2)	H8A—C8—H8C	109.5
O22—W5—O9	86.14 (19)	H8B—C8—H8C	109.5
O21—W5—O9	89.71 (19)	N3—C9—H9A	109.5
O30—W5—O9	164.60 (19)	N3—C9—H9B	109.5
O31—W5—O14	172.79 (19)	H9A—C9—H9B	109.5
O22—W5—O14	84.89 (17)	N3—C9—H9C	109.5
O21—W5—O14	73.96 (17)	H9A—C9—H9C	109.5
O30—W5—O14	82.76 (17)	H9B—C9—H9C	109.5
O9—W5—O14	82.39 (17)	C10—O35—Mn1	121.5 (5)
O23—W6—O24	98.7 (2)	O35—C10—N4	123.9 (7)
O23—W6—O22	101.4 (2)	O35—C10—H10	118.1
O24—W6—O22	89.35 (19)	N4—C10—H10	118.1
O23—W6—O17	100.9 (2)	C10—N4—C11	119.2 (7)
O24—W6—O17	91.0 (2)	C10—N4—C12	121.6 (6)
O22—W6—O17	157.46 (19)	C11—N4—C12	118.5 (7)
O23—W6—O4	97.0 (2)	N4—C11—H11A	109.5
O24—W6—O4	164.03 (19)	N4—C11—H11B	109.5
O22—W6—O4	84.77 (19)	H11A—C11—H11B	109.5
O17—W6—O4	88.8 (2)	N4—C11—H11C	109.5
O23—W6—O16	174.7 (2)	H11A—C11—H11C	109.5
O24—W6—O16	83.35 (18)	H11B—C11—H11C	109.5
O22—W6—O16	83.51 (17)	N4—C12—H12A	109.5
O17—W6—O16	74.15 (17)	N4—C12—H12B	109.5
O4—W6—O16	81.25 (17)	H12A—C12—H12B	109.5
O25—W7—O18	102.2 (2)	N4—C12—H12C	109.5
O25—W7—O24^i^	99.1 (2)	H12A—C12—H12C	109.5
O18—W7—O24^i^	87.7 (2)	H12B—C12—H12C	109.5
O25—W7—O5	98.0 (2)	C13—O36—Mn1	132.0 (6)
O18—W7—O5	85.6 (2)	O36—C13—N5	125.7 (8)
O24^i^—W7—O5	162.62 (19)	O36—C13—H13	117.1
O25—W7—O17	100.2 (2)	N5—C13—H13	117.1
O18—W7—O17	157.6 (2)	C13—N5—C15	120.4 (8)
O24^i^—W7—O17	89.6 (2)	C13—N5—C14	122.2 (8)
O5—W7—O17	90.5 (2)	C15—N5—C14	117.3 (8)
O25—W7—O16	173.4 (2)	N5—C14—H14A	109.5
O18—W7—O16	84.35 (18)	N5—C14—H14B	109.5
O24^i^—W7—O16	82.06 (18)	H14A—C14—H14B	109.5
O5—W7—O16	81.33 (17)	N5—C14—H14C	109.5
O17—W7—O16	73.26 (17)	H14A—C14—H14C	109.5
O26—W8—O18	102.1 (2)	H14B—C14—H14C	109.5
O26—W8—O30^i^	98.8 (2)	N5—C15—H15A	109.5
O18—W8—O30^i^	89.7 (2)	N5—C15—H15B	109.5
O26—W8—O19	100.6 (2)	H15A—C15—H15B	109.5
O18—W8—O19	157.2 (2)	N5—C15—H15C	109.5
O30^i^—W8—O19	89.3 (2)	H15A—C15—H15C	109.5
O26—W8—O6	98.3 (2)	H15B—C15—H15C	109.5
O18—W8—O6	85.8 (2)	C16—O37—Mn2	135.4 (7)
O30^i^—W8—O6	162.9 (2)	O37—C16—N6	125.2 (9)
O19—W8—O6	88.49 (19)	O37—C16—H16	117.4
O26—W8—O15	173.1 (2)	N6—C16—H16	117.4
O18—W8—O15	84.77 (18)	C16—N6—C18	119.5 (8)
O30^i^—W8—O15	81.74 (17)	C16—N6—C17	122.9 (8)
O19—W8—O15	72.53 (17)	C18—N6—C17	117.5 (8)
O6—W8—O15	81.44 (18)	N6—C17—H17A	109.5
O27—W9—O19	100.0 (2)	N6—C17—H17B	109.5
O27—W9—O28	98.7 (2)	H17A—C17—H17B	109.5
O19—W9—O28	89.7 (2)	N6—C17—H17C	109.5
O27—W9—O20	102.7 (2)	H17A—C17—H17C	109.5
O19—W9—O20	157.30 (19)	H17B—C17—H17C	109.5
O28—W9—O20	87.7 (2)	N6—C18—H18A	109.5
O27—W9—O7	97.4 (2)	N6—C18—H18B	109.5
O19—W9—O7	90.2 (2)	H18A—C18—H18B	109.5
O28—W9—O7	163.7 (2)	N6—C18—H18C	109.5
O20—W9—O7	86.1 (2)	H18A—C18—H18C	109.5
O27—W9—O15	172.9 (2)	H18B—C18—H18C	109.5
O19—W9—O15	72.92 (17)	C19—O38—Mn2	122.6 (8)
O28—W9—O15	82.15 (18)	O38—C19—N7	123.8 (11)
O20—W9—O15	84.38 (17)	O38—C19—H19	118.1
O7—W9—O15	82.27 (17)	N7—C19—H19	118.1
O16—P1—O15	112.7 (2)	C19—N7—C20	127.1 (11)
O16—P1—O14	111.5 (2)	C19—N7—C21	116.9 (10)
O15—P1—O14	111.9 (3)	C20—N7—C21	115.8 (11)
O16—P1—O13	107.1 (2)	N7—C20—H20A	109.5
O15—P1—O13	106.6 (3)	N7—C20—H20B	109.5
O14—P1—O13	106.5 (2)	H20A—C20—H20B	109.5
O37^ii^—Mn2—O37	180.0	N7—C20—H20C	109.5
O37^ii^—Mn2—O38^ii^	89.4 (3)	H20A—C20—H20C	109.5
O37—Mn2—O38^ii^	90.6 (3)	H20B—C20—H20C	109.5
O37^ii^—Mn2—O38	90.6 (3)	N7—C21—H21A	109.5
O37—Mn2—O38	89.4 (3)	N7—C21—H21B	109.5
O38^ii^—Mn2—O38	180.0	H21A—C21—H21B	109.5
O37^ii^—Mn2—O39^ii^	84.2 (3)	N7—C21—H21C	109.5
O37—Mn2—O39^ii^	95.8 (3)	H21A—C21—H21C	109.5
O38^ii^—Mn2—O39^ii^	90.4 (3)	H21B—C21—H21C	109.5
O38—Mn2—O39^ii^	89.6 (3)	C22—O39—Mn2	147.2 (9)
O37^ii^—Mn2—O39	95.8 (3)	O39—C22—N8	125.7 (14)
O37—Mn2—O39	84.2 (3)	O39—C22—H22	117.2
O38^ii^—Mn2—O39	89.6 (3)	N8—C22—H22	117.2
O38—Mn2—O39	90.4 (3)	C22—N8—C24	129.8 (14)
O39^ii^—Mn2—O39	180.0	C22—N8—C23	117.1 (14)
O34—Mn1—O32	91.0 (2)	C24—N8—C23	112.9 (13)
O34—Mn1—O36	96.2 (3)	N8—C23—H23A	109.5
O32—Mn1—O36	86.9 (2)	N8—C23—H23B	109.5
O34—Mn1—O35	89.2 (2)	H23A—C23—H23B	109.5
O32—Mn1—O35	169.7 (2)	N8—C23—H23C	109.5
O36—Mn1—O35	82.9 (2)	H23A—C23—H23C	109.5
O34—Mn1—O33	88.5 (3)	H23B—C23—H23C	109.5
O32—Mn1—O33	90.2 (2)	N8—C24—H24A	109.5
O36—Mn1—O33	174.5 (3)	N8—C24—H24B	109.5
O35—Mn1—O33	100.0 (2)	H24A—C24—H24B	109.5
O34—Mn1—O31	173.6 (2)	N8—C24—H24C	109.5
O32—Mn1—O31	94.1 (2)	H24A—C24—H24C	109.5
O36—Mn1—O31	88.0 (2)	H24B—C24—H24C	109.5
O35—Mn1—O31	86.5 (2)	O40—C25—N9	127.3 (17)
O33—Mn1—O31	87.5 (2)	O40—C25—H25	116.4
W1—O1—W2	123.2 (2)	N9—C25—H25	116.4
W2—O2—W3	123.7 (2)	C25—N9—C27	124.5 (19)
W3—O3—W1	123.7 (2)	C25—N9—C26	121.9 (14)
W1—O4—W6	151.7 (3)	C27—N9—C26	113.6 (18)
W2—O5—W7	152.0 (3)	N9—C26—H26A	109.5
W2—O6—W8	151.4 (3)	N9—C26—H26B	109.5
W3—O7—W9	150.4 (3)	H26A—C26—H26B	109.5
W3—O8—W4	151.3 (3)	N9—C26—H26C	109.5
W1—O9—W5	150.3 (3)	H26A—C26—H26C	109.5
P1—O13—W1	124.6 (3)	H26B—C26—H26C	109.5
P1—O13—W2	124.9 (2)	N9—C27—H27A	109.5
W1—O13—W2	90.64 (15)	N9—C27—H27B	109.5
P1—O13—W3	125.1 (2)	H27A—C27—H27B	109.5
W1—O13—W3	90.47 (15)	N9—C27—H27C	109.5
W2—O13—W3	90.57 (16)	H27A—C27—H27C	109.5
P1—O14—W5	128.5 (2)	H27B—C27—H27C	109.5
P1—O14—W4	127.9 (2)		

Symmetry codes: (i) −*x*+1, *y*, −*z*+1/2; (ii) −*x*+1/2, −*y*−1/2, −*z*.

### Hydrogen-bond geometry (Å, º)

**Table:**

*D*—H···*A*	*D*—H	H···*A*	*D*···*A*	*D*—H···*A*
C1—H1···O34	0.93	2.58	3.122 (10)	118
C2—H2*C*···O29^iii^	0.96	2.61	3.500 (12)	154
C4—H4···O21	0.93	2.53	3.431 (11)	163
C4—H4···O31	0.93	2.52	3.108 (10)	121
C8—H8*A*···O2^iv^	0.96	2.55	3.488 (14)	167
C8—H8*B*···O10^v^	0.96	2.57	3.307 (14)	134
C9—H9*A*···O1^iv^	0.96	2.60	3.366 (12)	137
C9—H9*B*···O35	0.96	2.42	3.289 (12)	151
C12—H12*A*···O23	0.96	2.48	3.439 (9)	176
C12—H12*C*···O11	0.96	2.48	3.086 (10)	121
C13—H13···O19^v^	0.93	2.53	3.287 (9)	139
C14—H14*B*···O30^iii^	0.96	2.56	3.379 (11)	144
C16—H16···O10	0.93	2.62	3.473 (11)	153
C17—H17*C*···O10	0.96	2.63	3.513 (12)	154
C19—H19···O40^ii^	0.93	2.43	3.331 (19)	164
C21—H21*B*···O11^iv^	0.96	2.59	3.478 (14)	154
C21—H21*C*···O33	0.96	2.50	3.375 (14)	151
C24—H24*C*···O26^vi^	0.96	2.43	3.369 (14)	164
C25—H25···O12^vii^	0.93	2.56	3.358 (19)	144
C26—H26*C*···O1^viii^	0.96	2.55	3.453 (17)	156

Symmetry codes: (ii) −*x*+1/2, −*y*−1/2, −*z*; (iii) −*x*+1/2, *y*+1/2, −*z*+1/2; (iv) −*x*+1/2, −*y*+1/2, −*z*; (v) *x*−1/2, *y*+1/2, *z*; (vi) *x*−1/2, *y*−1/2, *z*; (vii) −*x*+1, −*y*, −*z*; (viii) *x*, *y*−1, *z*.
